# Dual mediating effects of anxiety to use and acceptance attitude of artificial intelligence technology on the relationship between nursing students’ perception of and intention to use them: a descriptive study

**DOI:** 10.1186/s12912-024-01887-z

**Published:** 2024-03-28

**Authors:** Kyong Ah Cho, Yon Hee Seo

**Affiliations:** 1 Department of Nursing, Gwangju University, 277, Hyodeok-ro, Nam-gu, GwangJu, South Korea; 2 Department of Nursing, Andong National University, 1375, Gyeongdong-ro, Andong-si, Gyeongsangbuk-do South Korea

**Keywords:** Artificial Intelligence, AI, Perception, Intention to use AI, Nursing student

## Abstract

**Background:**

Artificial intelligence (AI)-based healthcare technologies are changing nurses’ roles and enhancing patient care. However, nursing students may not be aware of the benefits, may not be trained to use AI-based technologies in their practice, and could have ethical concerns about using them. This study was conducted to identify the dual mediating effects of anxiety to use and acceptance attitude toward AI on the relationship between perception of and intentions to use AI among nursing students in South Korea.

**Methods:**

The research model followed the PROCESS Macro model 6 proposed by Hayes. The participants were 180 nursing students in Gyeonggi-do. Data were collected from January 5–16, 2023, using self-reported questionnaires. Data were analyzed using the SPSS/WIN 25.0 program, with independent t-tests, one-way analysis of variance, Pearson’s correlations, and Hayes’s PROCESS macro method for mediation.

**Results:**

AI perception positively correlated with acceptance attitude (*r* =.44, *p <.*001), intention to use AI (*r* =.38, *p* <.001) and negatively correlated with anxiety (*r* = −.27, *p <.*001). Anxiety about AI negatively correlated with an acceptance attitude toward AI (*r* = −.36, *p <.*001) and intentions to use AI (*r* = −.28, *p <.*001). Acceptance attitude toward AI positively correlated with intentions to use AI (*r* =.43, *p <.*001). Anxiety about AI and acceptance attitude toward AI had a dual mediating effect on the relationship between AI perception and intentions to use AI.

**Conclusions:**

It is necessary to develop systematic educational programs to improve the perception of AI. Thus, the competency and professionalism of nursing students regarding the use of AI in healthcare can be improved.

## Background

Artificial intelligence (AI) is a collective term for technologies that train computers to emulate human cognitive functions, such as inference, communication, learning, and decision-making. Areas of AI include robotic engineering, machine learning, deep learning, and natural language processing, and most of these technologies have immediate relevance to the healthcare field, but the specific processes and tasks they support vary widely [1]. In nursing, AI robotic systems can not only mitigate the burdens associated with routine and repetitive tasks, such as the measurement of vital signs, measuring intake and outputs, and position changing [2] but also establish a streamlined nursing system [2, 3]. AI-based technology enhances nurses’ practical competencies and improves patient health outcomes [4].

AI-based technology will revolutionize nursing and healthcare by enhancing health promotion and disease prevention, facilitating the development of personalized treatment plans, automating tasks, and fostering collaboration among healthcare professionals [5]. Surprisingly, in the United States, a leader in advanced health technologies, 70% of nurses were unaware of AI-based technologies used in healthcare [5], and only 3.8% of nursing students received AI-related education during their undergraduate studies [6]. Moreover, previous studies [7, 8] showed that relatively few studies assess nurses’ perceptions or acceptance of the novel technology in healthcare. Although nurses and nursing students have high expectations for the usefulness and efficiency of AI-based technology in healthcare, no research has been done to identify how they perceive, feel about, or intend to use AI-based technology in the nursing field.

Nurses and nursing students’ perceptions, acceptance, and use of the novel technology are particularly important because of (a) the variety of systems, including AI-based technology in healthcare, used by nurses [2, 3] and (b) Nurses are key healthcare providers in the patient care [9, 10]. Potential users of AI-based technologies, nurses, and nursing students are uniquely positioned to influence and spearhead the application of AI in the nursing industry [11]. Thus, it is essential to show how users comprehend and adopt new technology, such as AI-based technology in healthcare [6].

The technology acceptance model (TAM) is one of the most popular research models to predict use regarding acceptance of the novel technology and intention to use it in specific healthcare [12], such as nurses [13] and patients [14]. The TAM presents perceived utility, perceived ease of use, and attitudes toward the new technology as predictors of the intent to use. According to Davis et al. [15], intention to use is directly related to an individual’s attitude. Intention to use is also related to perceived usefulness and ease of use. Attitude is predicted by perceived usefulness, and perceived usefulness is predicted by perceived ease of use in the novel technology. TAM posits that the perception of the novel technology leads to its acceptance, which results in actual use [16].

Emotion was an important predictor of risk perception and intention to use new technologies, such as AI-based technology [17]. Without expert knowledge of AI-based technology in healthcare, nursing students’ emotions, such as negative perceptions and anxiety regarding using AI-based technology, could be important mediating factors in their judgment of whether to use AI-based technology. However, extant studies have not verified that the perception of the AI-based technology has a mediating function in the relationship between psychological variables such as anxiety and intention to use the AI-based technology. Therefore, this study examined the mediating effects of emotions such as anxiety about AI and attitude of acceptance on the relationship between perception of and intention to use AI technologies among nursing students. The findings will help promote acceptance of AI technologies among nursing students, who are pivotal in the fourth industrial revolution and will present foundational data for more expansive research on perceptions toward AI.

## Methods

### Design

This was a descriptive study to identify the dual mediating effects of anxiety and acceptance attitude of AI on the relationship between factors affecting nursing students’ perception of and intention to use AI technology. The study model was designed based on Hayes’s PROCESS macro model 6.

### Participants

Participants from two universities located in two regions of South Korea were recruited using convenience sampling. The participants comprised students in the second to fourth year in nursing school in Gyeonggi province and Gwangju Metropolitan City, South Korea, with the following selection criteria: (1) they understand the purpose of this study and provide written consent for participation, (2) they have practicum experiences that are within the course of nursing science. We obtained permission for participant recruitment from the head of the nursing school from the two universities in G Province and Gwangju Metropolitan City, South Korea. We posted a recruitment note on the nursing notice board on the university homepage for two weeks. Then, Google Forms was used to distribute and collect the questionnaires. Participants were instructed to complete a self-report questionnaire after providing informed consent for the study online. Data were collected from January 5 to January 16, 2023. The sample size was determined using G*Power software (version 3.1.7, Heinrich-Heine University, Germany). For the regression analysis, based on the effect size of 0.15, significance level (⍺) of 0.05, statistical power (1-β) of 0.90, and nine arbitrary predictors (five general characteristics, perception of AI, anxiety about AI, acceptance attitudes toward AI, and intention to use AI), the minimum sample size was calculated as 141. We distributed the questionnaires to 183 students to account for dropouts. After excluding three insincere responses, we had 180 questionnaires in the final analysis (dropout rate = 1.6%).

### Measurements

#### Perception of AI

We measured the perception of AI using the Shinners Artificial Intelligence Perception tool developed by Shinners et al. [18]. It comprises ten items in two categories (preparedness for AI and professional impact of AI). We used a five-point Likert scale from 1 = “strongly disagree” to 5 = “strongly agree.” The higher the score, the higher the positive perception of AI. Cronbach’s α for preparedness for AI was 0.63, and for professional impact of AI was 0.83 in the original study [15] and 0.65 and 0.74, respectively, in this study.

#### Anxiety about AI

We used items from the Technology Acceptance Model (TAM) developed by Venkatesh et al. [19] to measure anxiety. We revised the term “technology” and/or “system” to “AI-based technology within the healthcare setting” in this study. The scale comprises four items on anxiety regarding the use of AI-based technologies. We used a five-point Likert scale (1 = “strongly disagree” to 5 = “strongly agree”). The higher the score, the higher the anxiety level regarding using AI-based technology. Cronbach’s α of the tool was 0.87 in a previous study [16] and 0.87 in this study.

#### Acceptance attitude toward AI

We used items from the TAM [19] to measure acceptance attitudes toward AI-based technologies. The tool comprises four items on acceptance attitudes toward AI-based technologies, and participants rated each item on a five-point Likert scale (1 = “strongly disagree” to 5 = “strongly agree”). The higher the score, the higher the level of acceptance attitude toward. Cronbach’s α of the tool was 0.82 in a previous study [19] and 0.90 in this study.

#### Intention to use the AI

We used items from the TAM [19] to measure intention to use AI-based technology. The tool comprises three items on intention to use AI-based technology, and participants rated each item on a five-point Likert scale (1 = “strongly disagree” to 5 = “strongly agree”). The higher the score, the higher the intention to use AI. Cronbach’s α of the tool was 0.69 in a previous study [19] and 0.68 in this study.

#### Ethical considerations

The study was approved by the Institutional Review Board of Gwangju University (no. 2-1041318-A-N-01-202211-HR-027-01). Written informed consent was obtained from all participants after the purpose and procedure of this study were explained to them. Participants were assured that there would be no disadvantages if they did not participate. The questionnaires were completed anonymously, and no personally identifiable information was collected.

#### Data analysis

Data analysis was performed using SPSS Statistics for Windows, version 26.0 (SPSS Inc., Chicago, Ill., USA). Participants’ general characteristics were analyzed using frequency analysis and descriptive statistics. The measured variable scores according to general characteristics were analyzed using t-tests and one-way analysis of variance. A post-hoc test was performed with an equivalence test followed by Scheffé’s. The correlation between variables was analyzed using Pearson’s correlation coefficient. SPSS PROCESS Macro model 6 [20] was used to test the mediating effect of anxiety and acceptance attitude toward AI on the relationship between participants’ perception of and intention to use AI. To verify the significance of the mediating effect, 10,000 bootstrap samples were extracted at a 95% confidence interval (CI). Bootstrapping reduces errors in the existing Sobel verification and does not require a large sample or the assumption of independence of path coefficients; therefore, it is widely used to verify mediating effects [21]. Before testing the mediating effect, multicollinearity between independent variables was confirmed using tolerance and variance inflation factor (VIF), and autocorrelation of dependent variables was confirmed using Durbin–Watson.

## Results

### Differences in variables by demographic characteristics

Participants were 154 women (85.6%, M_age_ = 22.10). Most were in the second year (80; 44.4%), followed by in the third (54; 30.0%) and fourth year (46; 25.6%). The group included 50 students (27.8%) with AI-related education. Analysis of the measured variables according to demographic characteristics showed a significant difference according to sex: women had higher anxiety than men (t = 4.064, *p <.*001).

According to grade, second-year students had a significantly higher perception of AI (F = 6.510, *p =.*002) than third- and fourth-year students. Second-year students had a significantly higher acceptance attitude toward AI than third-year students (F = 4.591, *p =.*011). The students with AI education experience had a significantly higher perception of AI (t = 2.076, *p =.*039) and acceptance attitude toward AI (t = 3.427, *p =.*001) and significantly lower anxiety (t = -2.715, *p =.*007) than those without an AI education. The t-tests and one-way analysis of variance analysis were conducted to measure the difference in AI use intention according to demographic variables such as gender, grade, and AI education experience of the study subjects. However, the difference was not significant, so this study did not consider differences between groups in AI use intention (Table 1).

**Table 1:** Differences in measured variables by demographic characteristics (*N* = 180)

Characteristic	Category	n (%) or M ± SD	Perception of AI		Anxiety about AI		Acceptance attitudes toward AI		Intention to use AI	
			M ± SD	t or F (p)	M ± SD	t or F (p)	M ± SD	t or F (p)	M ± SD	t or F (p)
Age (years)		22.10 ± 1.04								
Sex	Men	26 (14.4)	3.03 ± 0.54	0.055 (0.956)	2.69 ± 0.82	4.064 (< 0.001)	3.43 ± 0.92	1.266 (0.207)	3.41 ± 0.72	1.542 (0.126)
	Women	154 (85.6)	3.03 ± 0.53		3.37 ± 0.78		3.64 ± 0.77		3.63 ± 0.65	
Grade	Year 2^a^	80 (44.4)	3.19 ± 0.43	6.510 (0.002) b, c < a	3.15 ± 0.80	1.606 (0.204)	3.80 ± 0.72	4.591 (0.011) b < a	3.65 ± 0.62	0.823 (0.441)
	Year 3^b^	54 (30.0)	2.89 ± 0.58		3.39 ± 0.94		3.41 ± 0.90		3.60 ± 0.68	
	Year 4^c^	46 (25.6)	2.94 ± 0.58		3.34 ± 0.69		3.53 ± 0.72		3.49 ± 0.71	
AI education experience	Yes	50 (27.8)	3.16 ± 0.49	2.076 (0.039)	3.01 ± 0.84	-2.715 (0.007)	3.93 ± 0.69	3.427 (0.001)	3.73 ± 0.70	1.755 (0.081)
	No	130 (72.2)	2.98 ± 0.54		3.38 ± 0.79		3.49 ± 0.79		3.54 ± 0.64	

M = mean; SD = standard deviation; AI = artificial intelligence

### Perception, anxiety, acceptance attitude, and intention to use AI

The average scores (out of 5) of participants’ perception of AI were 3.03 ± 0.53; anxiety about AI, 3.27 ± 0.82; acceptance attitude toward AI, 3.61 ± 0.79; and intention to use AI, 3.59 ± 0.66 (Table 2).

**Table 2:** Descriptive statistics of measured variables (*N* = 180)

Variable	Range	Min	Max	M ± SD
Perception of AI	1–5	1.50	4.40	3.03 ± 0.53
Anxiety about AI	1–5	1.00	5.00	3.27 ± 0.82
Acceptance attitude toward AI	1–5	1.00	5.00	3.61 ± 0.79
Intention to use AI	1–5	2.00	5.00	3.59 ± 0.66

AI = artificial intelligence; M = mean; SD = standard deviation

### Correlations between perception, anxiety, acceptance attitude, and intention to use AI

Table 3 shows the correlations between perception, anxiety, acceptance attitude toward AI, and intention to use AI. Perception had a significant positive correlation with acceptance attitude toward AI (*r* =.44, *p <.*001), Intention to use AI (*r* =.38, *p* <.001), and a significant negative correlation with anxiety about AI (*r* = −.27, *p <.*001). Anxiety about AI had significant negative correlations with acceptance attitude toward AI (*r* = −.36, *p <.*001) and intention to use AI (*r* = −.28, *p <.*001). Acceptance attitude toward AI had a significant positive correlation with intention to use AI (*r* =.43, *p <.*001; Table 3).

**Table 3:** Correlations among measured variables (*N* = 180)

Variable	Perception of AI	Anxiety about AI	Acceptance attitude toward AI	Intention to use AI
	r (p)	r (p)	r (p)	r (p)
Perception of AI	1			
Anxiety about AI	− 0.27 (< 0.001)	1		
Acceptance attitude toward AI	0.44 (< 0.001)	− 0.36 (< 0.001)	1	
Intention to use AI	0.38(< 0.001)	− 0.28 (< 0.001)	0.43 (< 0.001)	1

AI = artificial intelligence

### The dual mediating effect of anxiety about AI and acceptance attitude toward AI

The assumptions of the regression analysis were verified before analyzing the mediating effects. The P-P plot was checked to determine the normality of the error term. The residuals were close to a 45º line, confirming normal distribution. The scatter plot of residuals revealed an even distribution around 0, confirming equal variance. The Durbin–Watson statistic was close to 2 at 2.240, suggesting the absence of autocorrelation among the residuals. The VIF was below 10, at 1.000–1.346, confirming the absence of multicollinearity. Thus, the study model satisfied all assumptions of the regression analysis—linearity of residuals, normality, equal variance, and independence.

To examine the mediating effects of anxiety about AI and acceptance attitude toward AI on the relationship between perception of and intention to use AI, analyses were performed using PROCESS Macro model 6. The model consisted of independent variables (X: perception of AI), a dependent variable (Y: intention to use AI), and two mediating variables (M1: anxiety about AI, M2: acceptance attitude toward AI).

Table 4 shows the direct effects of anxiety about AI and acceptance attitude toward AI on the relationship between perception of and intention to use AI. Perception of AI had a significant negative effect on anxiety about AI (b = − 0.420, *p* <.001). Perception of AI had a significant positive effect (b = 0.541, *p* <.001), while anxiety about AI had a significant negative effect (b = − 0.256, *p* <.001) on acceptance attitude toward AI. Perception of and acceptance attitude toward AI had significant positive effects (b = 0.280, *p* =.002; b = 0.246, *p* <.001, respectively) on the intention to use AI. To confirm the mediating effects of anxiety about AI and acceptance attitude toward AI, the effect of both the independent variable on the dependent variable and that of the independent variable on the mediator must be significant, and the effect of the independent variable on dependent variable must be reduced after adding the mediator [20]. In our study model, the effect of perception of AI on intention to use AI was reduced after adding anxiety about AI and acceptance attitude toward AI (b = 0.280, *p* =.002) compared to the effect without the mediator (b = 0.478, *p* <.001), confirming that anxiety and acceptance attitude toward AI are mediators in this relationship.

**Table 4:** Direct effects of anxiety and acceptance attitudes on the relationship between perception of and intention to use AI (*N* = 180)

Variable	Β	SE	t	*p*	LLCI	ULCI
					95% CI	95% CI
Perception of AI (X) → Anxiety about AI (M1)	− 0.420	0.111	3.773	< 0.001	− 0.639	− 0.200
	*R* =.272, R^2^ = 0.074, F = 14.233, *P* <.001					
Perception of AI (X) → Acceptance attitudes toward AI (M2)	0.541	0.100	5.424	< 0.001	0.344	0.738
Anxiety about AI (M1) → Acceptance attitudes toward AI (M2)	− 0.256	0.065	3.959	< 0.001	− 0.384	− 0.129
	*R* =.507, R^2^ = 0.257, F = 30.659, *P* <.001					
Perception of AI (X) → Intention to use AI (Y)	0.280	0.091	3.068	0.002	0.100	0.460
Anxiety about AI (M1) → Intention to use AI (Y)	− 0.091	0.057	1.593	0.113	− 0.204	0.022
Acceptance attitudes toward AI (M2) → Intention to use AI (Y)	0.246	0.064	3.856	< 0.001	0.120	0.371
	*R* =.495, R^2^ = 0.245, F = 19.076, *P* <.001					

AI = artificial intelligence; LLCI = lower level confidence interval; ULCI = upper level confidence interval; SE = standard error

**Fig. 1:**
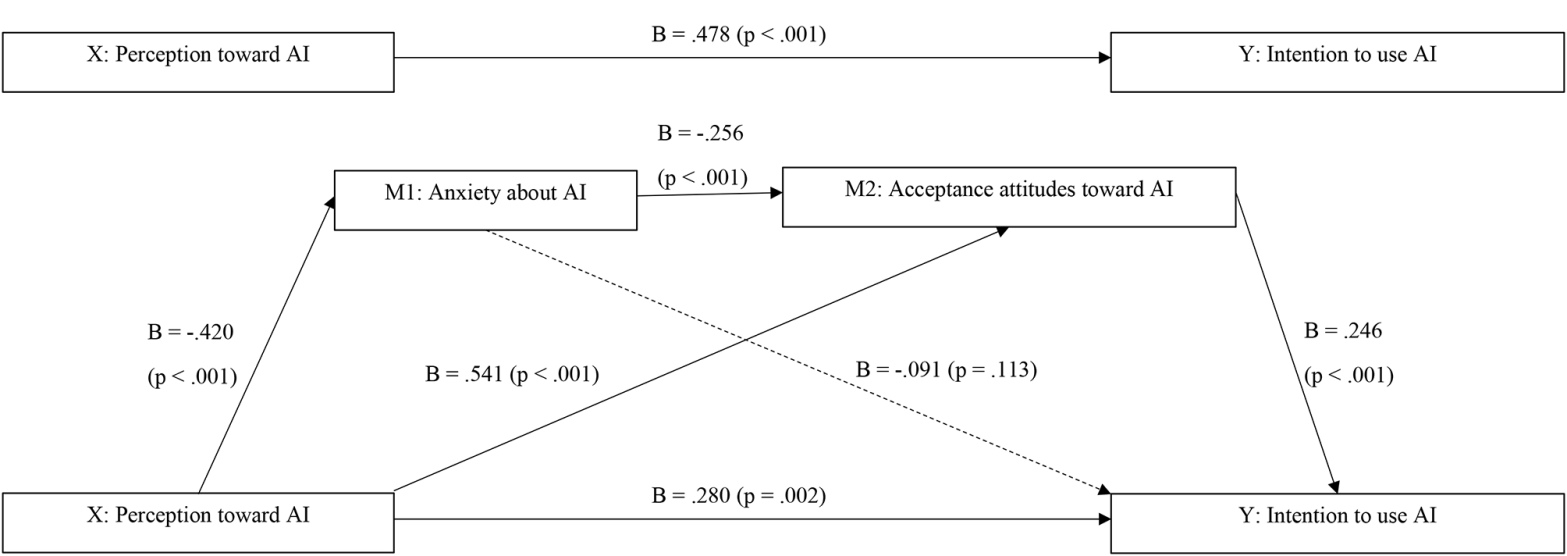
Direct effects of anxiety and acceptance attitudes on the relationship between perception of and intention to use AI AI = artificial intelligence

Table 5 shows the indirect effect of the independent variable on dependent variables through mediation by anxiety and acceptance attitude toward AI. The size of the overall mediating effect was 0.198 (95% CI [0.095, 0.332]), which was significant, as evidenced by the absence of 0 in the 95% bootstrap CI. In the analysis of simple mediating effects, the indirect effect size of anxiety about AI on the relationship between perception and intention to use (X → M1 → Y) was 0.038 (95% CI [-0.007, 0.098]), which was not significant as there was 0 in the 95% CI. The indirect effect size of acceptance attitude toward AI on the relationship between perception and intention to use (X → M2 → Y) was 0.133 (95% CI [0.047, 0.239]), which was significant as indicated by the absence of 0 in the 95% CI. In the dual mediating effect model, in which anxiety and acceptance attitude toward AI mediated the relationship between perception of and intention to use AI (X → M1 → M2 → Y), the effect size was 0.026 (95% CI [0.004, 0.066]); this was significant as evidenced by the absence of 0 in the 95% CI (Fig. 1).

**Table 5:** Indirect effects of anxiety and acceptance attitudes on the relationship between perception of and intention to use AI (*N* = 180)

Variable	Effect	Boot SE	Boot LLCI	Boot ULCI
			95% CI	95% CI
Perception of AI (X) → Anxiety about AI (M1) → Intention to use AI (Y)	0.038	0.027	− 0.007	0.098
Perception of AI (X) → Acceptance attitudes toward AI (M2) → Intention to use AI (Y)	0.133	0.049	0.047	0.239
Perception of AI (X) → Anxiety about AI (M1) → Acceptance attitudes toward AI (M2) → Intention to use AI(Y)	0.026	0.016	0.004	0.066
Total	0.198	0.058	0.095	0.322

AI = artificial intelligence; LLCI = lower level confidence interval; ULCI = upper level confidence interval; CI = confidence interval; SE = standard error

## Discussion

In this study, we delved into the mediating effects of anxiety about AI and acceptance attitude toward AI on the nexus between perception of AI and intention to use AI in nursing practice. We aimed to lay a foundation for initiatives that encourage the use of AI among nursing students by dissecting the interrelations among anxiety about AI [17], perception of AI, and acceptance attitude toward AI [22], all identified as influential in shaping the intent to use AI.

The average perception score of AI among nursing students was 3.03 ± 0.53, mirroring the findings in similar cohorts of healthcare professionals [18] and nursing students [22]. This resemblance suggests that the students’ expectations for AI in healthcare — particularly its potential to alleviate routine task burdens and enrich nursing care quality [2–4] — have significantly shaped their perceptions. The average anxiety level regarding AI clocked in at 3.27 ± 0.82, a figure akin to that reported in health major undergraduates [23]. Notably, only a fraction (27.8%) of our participants had been exposed to AI-related education. Prior research indicates a gap between the perceived need for AI education and the practical utility of current AI training in preparing students for AI adoption in healthcare [6, 24]. This educational gap might contribute to the heightened anxiety levels among nursing students, stemming from uncertainties about AI’s impact on their profession [4, 5].

In terms of acceptance attitude and intention to use AI, nursing students scored 3.61 ± 0.79 and 3.59 ± 0.66, respectively. These figures are higher than those observed among health major undergraduates [23]. The variability in attitudes towards AI adoption could be attributed to several factors, including age, gender, field-specific contexts of AI use [25], individual personality traits and cultural backgrounds [26]. Moreover, the relatively younger demographic in our study might explain the higher acceptance rates of AI, possibly due to a greater alignment with technology and innovation [25]. Additionally, research indicating nurses’ positive assessment of robotic systems in healthcare [3] and their high expectations of such systems to improve nursing care quality [2–4] might illuminate why nursing students strongly intend to use AI.

We also explored how anxiety about AI and acceptance attitude toward AI mediate the relationship between perception of AI and intention to use AI. We observed that a more favorable perception of AI correlates with reduced anxiety. This trend aligns with the notion that positive expectations and understanding AI’s role in healthcare can mitigate fears and apprehensions [19, 23]. Moreover, an enhanced perception of AI leads to a more accepting attitude towards its use.

This finding echoes previous research suggesting that greater awareness of AI’s practical applications in daily healthcare operations boosts its acceptance [27].

Furthermore, we found that an acceptance attitude toward AI plays a dual mediating role. Not only does it directly influence the intention to use AI, but it also does so indirectly by modulating anxiety levels. This underscores the importance of positive attitudes in fostering an intention to use AI in healthcare [6, 28]. Negative perceptions, such as viewing AI as a threat to job security, can conversely lead to a more negative acceptance attitude and, consequently, a decreased intention to use AI.

From the perspective of applying results in nursing education and clinical nursing practice, this study highlights the critical role of both in leveraging AI’s potential in healthcare. It stresses the need for comprehensive AI education within the nursing curriculum to close the educational gap and reduce anxiety about AI among nursing students. The educational framework should cover AI’s technical aspects and its practical healthcare applications to foster a positive view and acceptance attitude toward AI. Furthermore, clinical nursing practice can advance by promoting an AI-friendly culture, demonstrating AI’s successful use in routine nursing tasks and patient care to mitigate fears and enhance confidence in AI technologies. Through specific workshops, seminars, and hands-on sessions that showcase AI’s benefits, including workload reduction and improved care quality, a better understanding and acceptance attitude toward AI among nursing students and professionals can be achieved. This approach could lead to broader and more effective AI use in healthcare, resulting in better patient outcomes and more efficient nursing practices.

In conclusion, our findings emphasize the crucial role of educational interventions that enhance understanding and acceptance of AI among nursing students. Such initiatives can positively impact their intention to use AI in healthcare, thus potentially leading to improved healthcare outcomes.

Our study, however, is not without limitations. The cross-sectional design precludes us from drawing causal inferences. Future research, preferably longitudinal, should be conducted. Furthermore, the lack of direct experience with AI technologies among most participants points to the need for studies involving students who have had practical exposure to AI in healthcare settings.

## Conclusion

Perception, anxiety, and acceptance toward AI are important factors influencing the intention to use AI, and anxiety and acceptance have a dual mediating effect on the relationship between perception of and intention to use AI among nursing students. Nursing educational institutions should provide systematic AI-related education to improve nursing students’ perception of AI, reducing their anxiety about it and positively transforming their acceptance of AI, ultimately boosting their intention to use it. Therefore, there is a pressing need for nursing education programs that bolster AI competencies to cultivate nurses capable of proactively adapting to the rapidly evolving healthcare environment during the fourth industrial revolution.

## Data Availability

The datasets used and/or analyzed during this study are available from the corresponding author upon reasonable request.

## Abbreviations

AI: Artificial intelligence
TAM: Technology Acceptance Model
VIF: Variance inflation factor
CI: Confidence interval
